# Computer-assisted discrimination of cancerous and pre-cancerous from benign oral lesions based on multispectral autofluorescence imaging endoscopy

**DOI:** 10.1117/1.BIOS.1.2.025001

**Published:** 2024-07-03

**Authors:** Elvis de Jesus Duran Sierra, Shuna Cheng, Rodrigo Cuenca, Beena Ahmed, Jim Ji, Vladislav V. Yakovlev, Mathias Martinez, Moustafa Al-Khalil, Hussain Al-Enazi, Carlos Busso, Javier A. Jo

**Affiliations:** a Texas A&M University, Department of Biomedical Engineering, College Station, Texas, United States; b University of Oklahoma, School of Electrical and Computer Engineering, Norman, Oklahoma, United States; c University of New South Wales, School of Electrical Engineering and Telecommunications, Sydney, New South Wales, Australia; d Texas A&M University at Qatar, Department of Electrical and Computer Engineering, Doha, Qatar; e Hamad Medical Corporation, Department of Cranio-Maxillofacial Surgery, Doha, Qatar; f Hamad Medical Corporation, Department of Otorhinolaryngology Head and Neck Surgery, Doha, Qatar; g The University of Texas at Dallas, School of Electrical and Computer Engineering, Dallas, Texas, United States

**Keywords:** oral cancer, oral dysplasia, autofluorescence imaging, machine learning, optical imaging

## Abstract

**Significance:**

Diagnosis of cancerous and pre-cancerous oral lesions at early stages is critical for the improvement of patient care, to increase survival rates and minimize the invasiveness of tumor resection surgery. Unfortunately, oral precancerous and early-stage cancerous lesions are often difficult to distinguish from oral benign lesions with the existing diagnostic tools used during standard clinical oral examination. In consequence, early diagnosis of oral cancer can be achieved in only about 30% of patients. Therefore, clinical diagnostic technologies for fast, minimally invasive, and accurate oral cancer screening are urgently needed.

**Aim:**

This study investigated the use of multispectral autofluorescence imaging endoscopy for the automated and noninvasive discrimination of cancerous and precancerous from benign oral epithelial lesions.

**Approach:**

*In vivo* multispectral autofluorescence endoscopic images of clinically suspicious oral lesions were acquired from 67 patients undergoing tissue biopsy examination. The imaged lesions were classified as precancerous (*n* = 4), cancerous (*n* = 29), and benign (*n* = 34) lesions based on histopathology diagnosis. Multispectral autofluorescence intensity feature maps were generated for each oral lesion and used to train and optimize support vector machine (SVM) models for automated discrimination of cancerous and precancerous from benign oral lesions.

**Results:**

After a leave-one-patient-out cross-validation strategy, an optimized SVM model developed with four multispectral autofluorescence features yielded levels of sensitivity and specificity of 85% and 71%, respectively and overall accuracy of 78% in the discrimination of cancerous/precancerous versus benign oral lesions.

**Conclusion:**

This study demonstrates the potentials of a computer-assisted detection system based on multispectral autofluorescence imaging endoscopy for the early detection of cancerous and precancerous oral lesions.

## 1 Introduction

Early detection of new and recurrent oral cancer holds great promise for improving the survival rate and quality of life of patients. Unfortunately, precancerous and cancerous oral lesions are often difficult to distinguish from benign oral lesions during standard clinical examination.^1,2^ As a result, only about 30% of patients are diagnosed at early stages despite the fact that the oral cavity is easily accessible for direct examination.^3^ Therefore, there is an urgent need for novel clinical diagnostic technologies capable of improving the rate of early oral cancer detection.

Commercially available diagnostic adjuncts, including toluidine blue,^4^ brush cytology,^5^ acetowhitening with chemiluminescence (ViziLite),^6^ and autofluorescence imaging (VELscope, Identafi, and OralID),^7,8^ have been widely evaluated during clinical examination of potentially malignant and premalignant oral lesions. However, these diagnostic adjuncts have displayed low specificity and are not generally recommended for the assessment of clinically suspicious oral lesions.^9,10^

Optical imaging technologies have also been developed and evaluated for the early detection of oral cancer. Kozakai et al. performed autofluorescence imaging in 50 patients using the Illumiscan^®^ (SHOFU, Kyoto, Japan) fluorescence visualization device and reported levels of sensitivity and specificity of 85% and 93%, respectively in cancerous versus benign oral lesions.^11^ Wang et al. performed autofluorescence spectroscopy in 97 patients and reported 81% sensitivity and 96% specificity in the classification of pre-cancerous/cancerous versus benign/normal oral tissues.^12^ Guze et al. performed *in vivo* Raman spectroscopy in 18 patients to distinguish pre-cancerous and cancerous from benign and normal oral tissues and reported levels of sensitivity and specificity of 100% and 77%, respectively.^13^ Finally, Chen et al. differentiated precancerous versus benign oral lesions from 38 patients with 68% sensitivity and 95% specificity using in vivo time-resolved fluorescence spectroscopy.^14^ Although these studies have demonstrated the potentials of optical imaging technologies for the early detection of oral cancer, further research is needed to successfully translate these diagnostic systems into the clinic.

In a previous study conducted by our group,^15^ we demonstrated clinical widefield multispectral autofluorescence endoscopic imaging of novel biochemical and metabolic biomarkers of oral dysplasia and cancer, and their potential for early detection. These autofluorescence biomarkers are mainly associated to the endogenous fluorophores reduced-form nicotinamide adenine dinucleotide (NADH) and flavin adenine dinucleotide (FAD) in oral epithelial cells; and collagen in the underlying lamina propria. In this study, we hypothesized that these oral cancer biomarkers can be used as features to develop predictive machine learning models for the automated and non-invasive discrimination between cancerous/pre-cancerous and benign oral epithelial lesions.

## 2 Methods

### 2.1 Imaging System and Data Acquisition Protocol

The multispectral autofluorescence endoscope system used in this study was developed by Cheng et al.^16^ and is currently undergoing upgrades^17-19^ to improve the clinical imaging of established and potentially novel autofluorescence biomarkers of oral pre-malignancy and malignancy. The system consisted in a 355 nm pulsed laser (Advanced Optical Technology, 1 ns pulse width, ~1 *μ*J/pulse at the tissue), which collected the tissue autofluorescence emission at the 390 ± 20 nm, 452 ± 22.5 nm, and >500 nm spectral bands, selected to preferentially measure collagen, NADH, and FAD autofluorescence, respectively. The system delivered a total energy of 2.8 mJ into the patient’s oral mucosa, being an order of magnitude lower than the maximum permissible exposure (MPE = 29.8 mJ) provided by the American National Standards Institute.^20^ The autofluorescence endoscopic images were acquired with a 10 mm circular field-of-view, ~100 *μ*m lateral resolution, and <3 s acquisition time per multispectral image. Clinical examination of the patient’ s oral cavity was performed by an experienced head and neck surgeon (M.M., M.A.K., and H.A.E), followed by the acquisition of endoscopic multispectral autofluorescence images from both the suspicious oral lesion and a clinically healthy-appearing area in the corresponding contralateral anatomical side. The image acquisition protocol was approved by the Institutional Review Boards at Hamad Medical Corporation (Doha, Qatar). An incisional tissue biopsy was then taken from the center of the lesion, which was blinded to the autofluorescence endoscopy imaging acquisition and processing. Each imaged oral lesion was annotated based on its tissue biopsy histopathological diagnosis (gold standard). The imaged healthy appearing tissue area from the contralateral side of the lesion was not biopsied. The distribution of the 67 multispectral autofluorescence endoscopic images of benign, precancerous, and cancerous oral lesions acquired in this study is summarized in Table 1.

### 2.2 Multispectral Autofluorescence Image Pre-Processing

The following steps were applied to preprocess the autofluorescence endoscopic images: (1) the signal offset and background were subtracted at every pixel of the image, (2) pixels presenting signal saturation were masked by applying a threshold on the maximum signal amplitude, (3) a 5 × 5 spatial averaging filter was used to improve the signal-to-noise ratio (SNR) at every pixel, (4) pixels with an SNR value below 15 decibels were masked, and (5) additional pixels were manually masked from images containing teeth regions characterized by a strong autofluorescence emission.

### 2.3 Computation of Absolute Spectral Features

The multispectral autofluorescence endoscopic image dataset is composed of intensity temporal decay signals $y_{\lambda }(x,y,t)$, acquired at each emission band (λ) and spatial location (x,y). After all the multispectral autofluorescence images were pre-processed, autofluorescence intensity features were computed at every pixel. The multispectral absolute autofluorescence intensity $I_{\lambda }(x,y)$ was computed by numerically integrating the fluorescence intensity temporal decay signal using Eq. (1) and was then normalized using Eq. (2)

(1) $$I_{\lambda }(x,y)=\int y_{\lambda }(x,y,t)\mathrm{dt},$$

(2) $$I_{\lambda ,n}(x,y)=\frac{I_{\lambda }(x,y)}{\sum_{\lambda }I_{\lambda }(x,y)}.$$

From the multispectral absolute autofluorescence intensities, six spectral ratios were computed to quantify the relative intensity values between emission spectral channels: $I_{390}(x,y)∕I_{452}(x,y)$, $I_{390}(x,y)∕I_{500}(x,y)$, $I_{452}(x,y)∕I_{500}(x,y)$, $\left[I_{452}(x,y)+I_{500}(x,y)\right]∕I_{390}(x,y)$, $\left[I_{390}(x,y)+I_{500}(x,y)\right]∕I_{452}(x,y)$, and $\left[I_{390}(x,y)+I_{452}(x,y)\right]∕I_{500}(x,y)$.

The normalized autofluorescence intensities computed at the 390 ± 20 nm $\left(I_{390,n}\right)$, 452 ± 22.5 nm $\left(I_{452,n}\right)$, and >500 nm $\left(I_{500,n}\right)$ emission bands, primarily quantify the autofluorescence originated from the endogenous fluorophores collagen in the oral submucosa, and NADH and FAD within oral epithelial cells, respectively. The spectral ratio $I_{452}∕I_{500}$ is associated to the optical redox-ratio^21^ and quantifies the autofluorescence of NADH at the 452 ± 22.5 nm band relative to that of FAD at the >500 nm band. These features represent established morphologic and metabolic biomarkers of oral cancer.^15,22^ The remaining five spectral intensity ratios measure the relative autofluorescence emissions between collagen, NADH, and FAD and can potentially represent novel oral epithelial cancer biomarkers.^15^

### 2.4 Computation of Relative Spectral Features

Relative values Δf(x,y) for each spectral feature were computed as follows. First, absolute spectral feature maps (Sec. 2.3) were generated for both the lesion and paired healthy tissue images. Second, the difference between each pixel in the lesion feature map f(x,y) and the median value of the corresponding healthy feature map $\mu_{f,\mathrm{Healthy}}$ was computed for each spectral feature as shown in Eq. (3). The relative spectral features thus represent the result of normalizing the lesion image feature distribution with respect to the median of the healthy image feature distribution

(3) $$\Delta f(x,y)=f(x,y)-\mu_{f,\mathrm{Healthy}}.$$

In summary, a total of 18 autofluorescence spectral intensity features were computed per pixel, as summarized in Table 2.

### 2.5 Machine Learning Model Optimization and Classification Performance Estimation

**Fig. 1:**
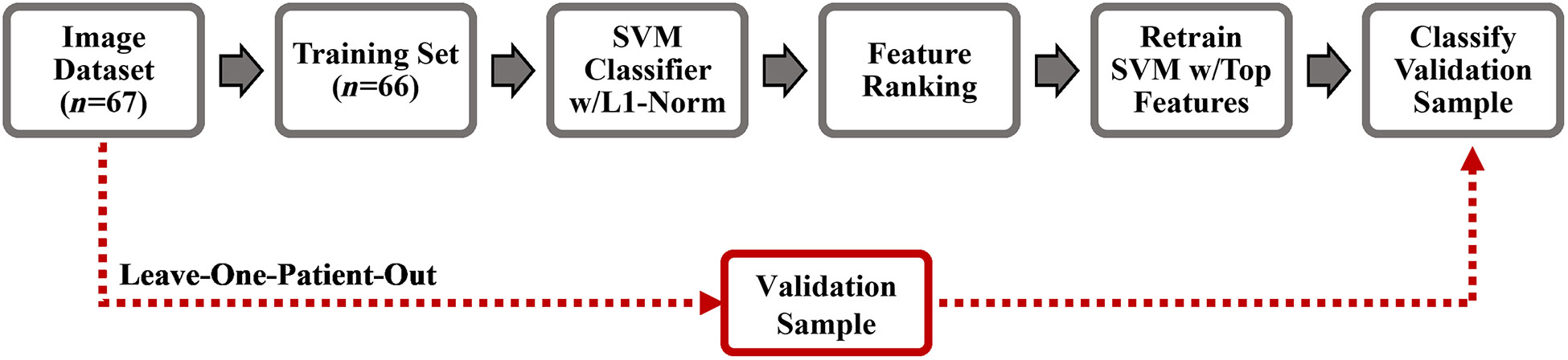
Schematic of the leave-one-patient-out-cross-validation process used to estimate the classification performance. This process was performed using the top three, four, and five features to retrain the L1-SVM model.

The classification performance for the discrimination of cancerous/pre-cancerous [squamous cell carcinoma (SCC), moderate and high-grade dysplasia; *n* = 33] from benign (*n* = 34) oral lesions was estimated as follows. The multispectral autofluorescence endoscopic image dataset of 67 oral lesion images was introduced into a classification model optimization process using a leave-one-patient-out-cross-validation (LOPOCV) strategy illustrated in Fig. 1. First, one lesion image from a patient was removed from the dataset and used as the validation sample, which was blinded to the model training. The remaining 66 images from all other patients were used as the training set. Second, the training set was introduced into a linear support vector machine (SVM) model with L1-regularization (L1-SVM; C=1),^23^ which assigned weights to each of the spectral features. The absolute value of each weight was taken, and the weights were normalized. The features were then ranked from largest to smallest weight. Third, to prevent overfitting due to the small sample size of the training set (*n* = 66), the L1-SVM model was retrained using only the features with largest weights and applied to the validation sample. Finally, the whole process was repeated until each of the 67 oral lesion images was used as the validation sample. This LOPOCV strategy was evaluated using the top three, four, and five features to retrain the L1-SVM model.

**Fig. 2:**
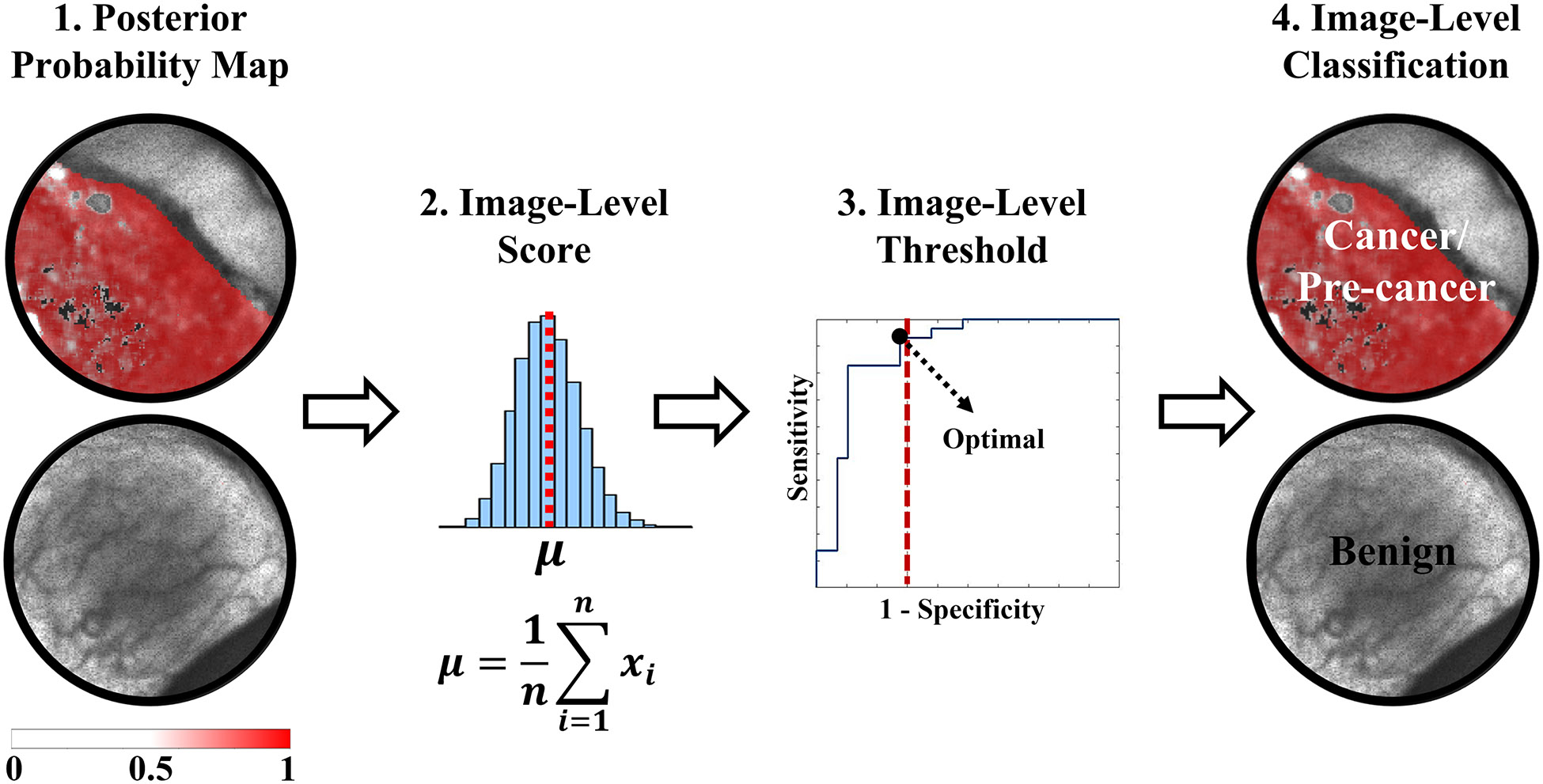
Summary of the image-level classification process performed in this study. (1) The L1-SVM posterior probability map was obtained from the pixel-level classification, (2) an image-level score consisting in the average of the posterior probability map was computed, (3) this score was compared against an optimized threshold from the training set, and (4) the image was classified as cancerous/pre-cancerous if the image-level score was greater than or equal to the threshold or as benign otherwise.

To classify the independent validation sample, the process illustrated in Fig. 2 was performed at every LOPOCV iteration: (1) a trained L1-SVM model was applied at the pixel-level resulting in a posterior probability map, in which every pixel value represents the likelihood of cancer/precancer, (2) an image-level score was generated by computing the average of the posterior probability map, (3) receiver operating characteristic (ROC) analysis was performed on the image-level scores from the training set, and an image-level score threshold was optimized by selecting the point on the ROC curve with maximum sensitivity within the 1-specificity range of 0% to 30%, and (4) finally, the validation sample was classified as cancer/precancer if its predicted image-level score was greater than or equal to the optimized threshold, or as benign otherwise.

After the LOPOCV process was completed, a confusion matrix was generated, and the image-level classification performance was quantified in terms of the levels of sensitivity and specificity using Eqs. (4) and (5), respectively:

(4) $$\text{Sensitivity}=\frac{\text{TP}}{\text{TP}\;+\;\text{FN}},$$

(5) $$\text{Specificity}=\frac{\text{TN}}{\text{TN}\;+\;\text{FP}},$$

where TP, FN, TN, and FP represent the number of true positives, false negatives, true negatives, and false positives, respectively.

### 2.6 Classification Model Simulating the VELscope Imaging System

The VELscope system is an autofluorescence-based commercially available clinical diagnostic adjunct that has been widely used for the early detection of oral cancer.^8^ It delivers blue light excitation (400 to 460 nm) to the oral mucosa, and pale green autofluorescence is associated to normal oral tissue, while dark autofluorescence to abnormal oral tissue. In this study, a linear L1-SVM model (C=1) using a single spectral feature consisting in the combined normalized autofluorescence intensities at the 452 ± 22.5 nm and >500 nm emission bands $\left(I_{452,n}+I_{500,n}\right)$, simulating the VELscope autofluorescence emission, was also trained and cross-validated using LOPOCV (Fig. 1). The LOPOCV classification performance (sensitivity and specificity) of the optimal L1-SVM model identified using multispectral autofluorescence features was compared against the performance of the single-feature L1-SVM model mimicking the VELscope.

## 3 Results

The classification results following the LOPOCV strategy for each spectral feature pool, and for the top three, four, and five spectral features selected to retrain the L1-SVM model are summarized in Table 3. The L1-SVM model developed with the top four features from the absolute spectral feature pool displayed the highest levels of sensitivity (85%) and specificity (71%) among all cases.

**Fig. 3:**
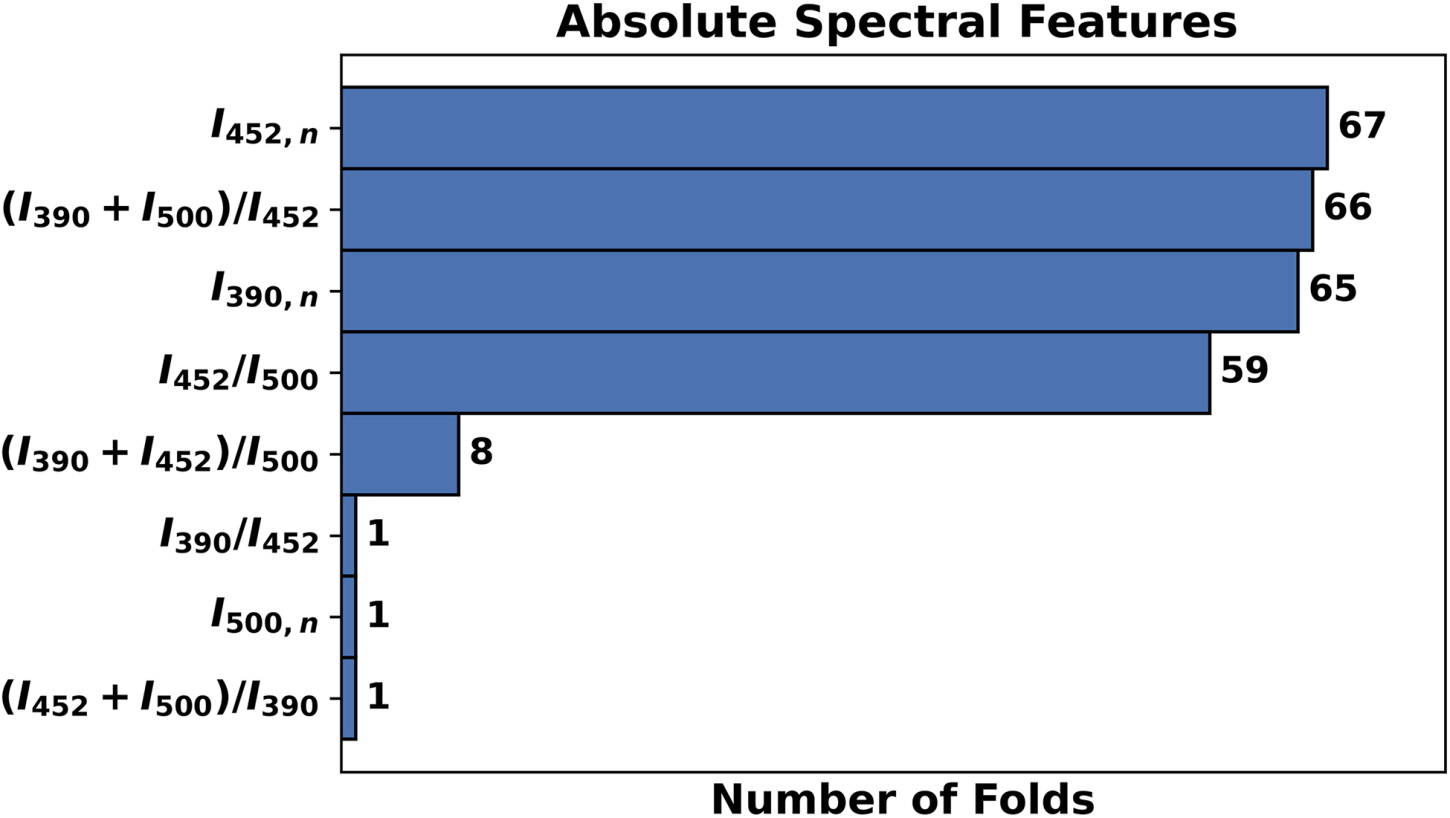
Frequency of absolute spectral features selected by the L1-SVM model retrained with the top 4 features.

After completion of the LOPOCV process, the frequency of the spectral features selected by the L1-SVM model was recorded for each feature pool evaluated. Figure 3 presents the frequency of the features selected by the best performing L1-SVM model (using the top four absolute spectral features, Table 3). The most relevant absolute spectral features identified included $I_{452,n}$ (selected in 100% of the folds), $\left[I_{390}+I_{500}\right]∕I_{452}$ (selected in 98% of the folds), $I_{390,n}$ (selected in 97% of the folds), and $I_{452}∕I_{500}$ (selected in 88% of the folds).

**Fig. 4:**
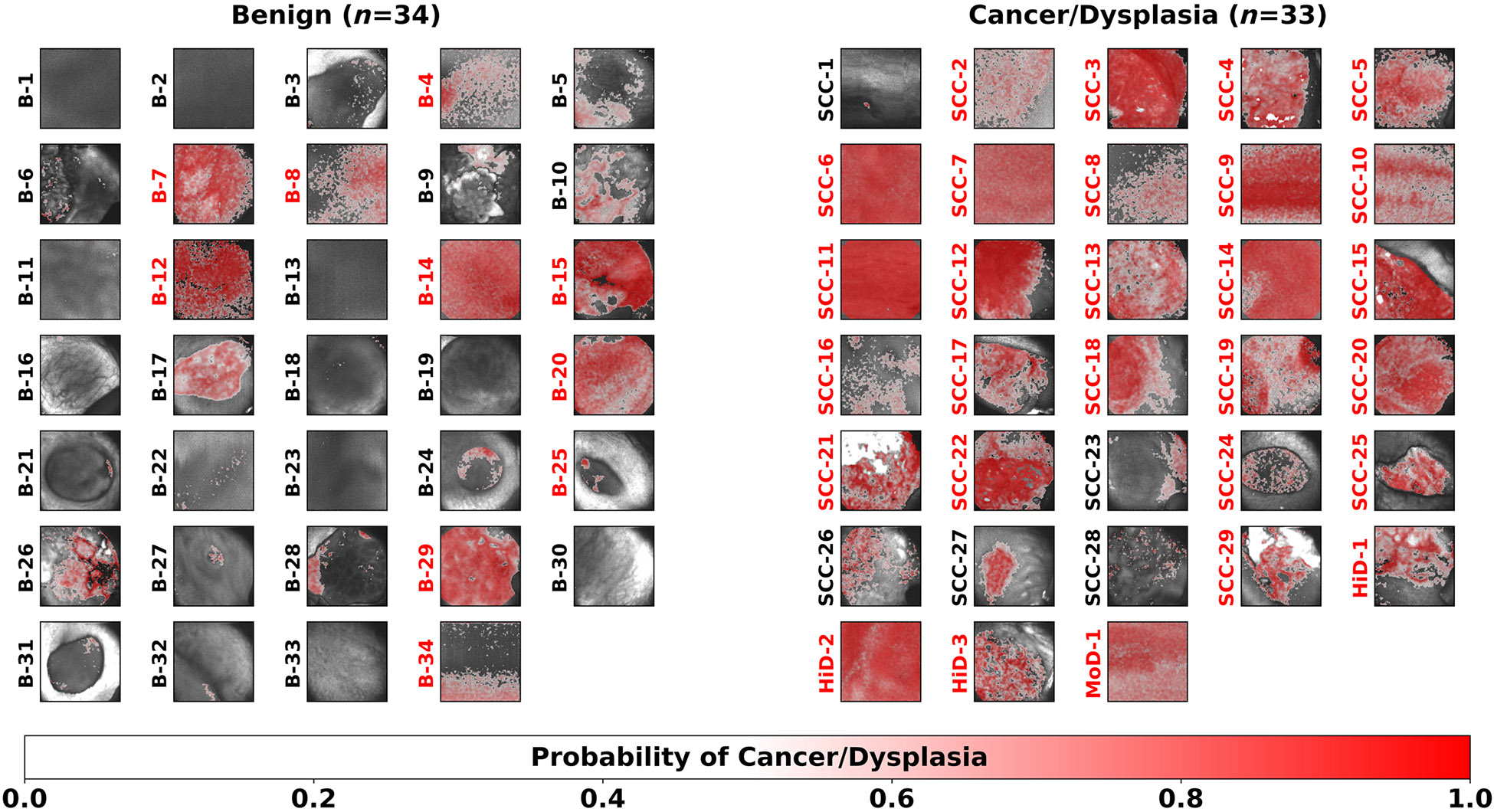
Posterior probability maps (red intensity scale) superposed on the total autofluorescence intensity images (gray intensity scale) of 67 oral lesions obtained from the best performing L1-SVM model (with top four features). Lesion identification labels are shown to the left of each map and color-coded in red if classified as cancer/precancer based on the image-level classification and in black otherwise.

Figure 4 presents the posterior probability maps of the 67 oral lesions resulting from the LOPOCV using the best performing L1-SVM model. In these maps, each pixel was color-coded based on the predicted probability, with red tones indicating higher likelihood of cancer/precancer.

The confusion matrices resulting from LOPOCV comparing the predicted oral lesion labels of the optimal model (L1-SVM with top four features) against the single-feature $\left(I_{452,n}+I_{500,n}\right)$ model simulating the VELscope are presented in Table 4. The L1-SVM with the top four features was able to correctly predict 24/29 cancerous (SCC), 4/4 pre-cancerous (3 HiD and 1 MoD), and 24/34 benign oral lesions, resulting in levels of sensitivity and specificity of 85% and 71%, respectively and overall accuracy of 78%, while the single-feature L1-SVM model achieved lower sensitivity (61%), same specificity (71%), and lower accuracy (66%).

## 4 Discussion

Multispectral autofluorescence endoscopic images of benign, pre-cancerous, and cancerous oral lesions from 67 patients undergoing tissue biopsy examination were acquired *in vivo*. For every imaged oral lesion, absolute and relative autofluorescence spectral feature maps were generated and used to optimize machine learning classification models for the automated discrimination of cancerous/precancerous versus benign oral lesions. Results from this study provide strong preliminary data supporting the potentials of multispectral autofluorescence imaging endoscopy for the early detection of oral cancer.

Among the three autofluorescence spectral feature pools used to develop the L1-SVM model, the absolute spectral features resulted critical in the discrimination of cancerous/precancerous from benign lesions since they provided the highest levels of sensitivity (85%) and specificity (71%).

One of the most relevant biomarkers identified in our analysis was the normalized autofluorescence intensity at the 390 ± 20 nm emission band $\left(I_{390,n}\right)$, mainly associated to collagen autofluorescence. This biomarker has been shown to decrease in malignant and premalignant oral lesions^15,22,24^ due to a breakdown of collagen crosslinks and increased epithelial thickness.^25,26^

Another important biomarker identified was the normalized autofluorescence intensity measured at the 452 ± 22.5 nm emission band $\left(I_{452,n}\right)$, mainly associated to NADH autofluorescence. The relevance of this feature in the discrimination of cancerous/precancerous from benign oral lesions might rely on the metabolic pathways used by neoplastic cells, since energy production in these cells is characterized by increased used of glycolysis and citric-acid (Krebs) cycle.^27^ These metabolic pathways reduce NAD+ into fluorescent NADH, resulting in increased concentration of mitochondrial NADH and higher tissue autofluorescence emission within the 452 ± 22.5 nm band $\left(I_{452,n}\right)$.

The optical redox-ratio $\left(I_{452}∕I_{500}\right)$ represented another relevant biomarker in the discrimination of cancerous/precancerous versus benign lesions. This feature has been shown to decrease in cancerous and precancerous oral lesions,^15,22,24^ likely reflecting increased cellular metabolic activity associated to higher activation of the oxidative phosphorylation pathway in malignant cells.^28^

The last relevant feature identified in this study was the ratio $\left[I_{390}+I_{500}\right]∕I_{452}$, which quantifies the combined autofluorescence of collagen at the 390 ± 20 nm band and FAD at the >500 nm band, with respect to NADH autofluorescence at the 452 ± 22.5 nm band. To the best of our knowledge, no previous studies have reported a trend in this feature in cancerous/precancerous oral lesions; thus, representing a potentially new autofluorescence biomarker of oral epithelial cancer.

Finally, the optimal classification model identified in this study was able to correctly classify a larger number of cancerous/precancerous oral lesions, resulting in higher sensitivity (85%), compared to the model simulating the VELscope system with 61% sensitivity. This finding highlights the advantage of our multispectral endoscopy system over a single emission wavelength system, since absolute spectral features derived from the autofluorescence acquired at three different emission bands were critical in the discrimination of cancerous/precancerous versus benign lesions. However, further validation is needed by performing a more realistic comparison against the actual VELscope imaging system. Nevertheless, these findings demonstrate the potentials of multispectral autofluorescence imaging endoscopy for early oral cancer screening in a clinical setting.

### 4.1 Study Limitations

This study used the average of the L1-SVM-derived posterior probability maps to summarize them into image-level scores that were compared against optimized thresholds to obtain the final image-level classification (cancerous/precancerous versus benign). However, other approaches, such as the distribution median and mode, and percentage of pixels above a fixed-probability threshold, could be used to summarize the posterior probability maps into image-level score representations. Future studies further validating these promising results will consider other alternatives that can potentially provide better single-score representations of the classifier posterior probability maps.

The limited specificity (71%) achieved in this study by the best performing L1-SVM model needs to be improved to enable successful clinical translatability of our multispectral autofluorescence endoscopic imaging system. To achieve this, our future research efforts will focus on (1) collecting data at multiple medical centers to generate a larger and more diverse oral lesion database that could improve the performance of our L1-SVM classifiers; and (2) the investigation of time-resolved autofluorescence features, such as the multispectral average autofluorescence lifetime and bi-exponential decay model parameters,^29^ which could provide complementary information to potentially improve the L1-SVM discrimination of cancerous/precancerous versus benign oral lesions.

Altogether, the established and potentially new autofluorescence biomarkers of oral cancer used as features in an L1-SVM classification model for the discrimination of cancerous/pre-cancerous versus benign oral lesions support the potentials of multispectral autofluorescence imaging endoscopy as a non-invasive clinical diagnostic tool for early detection of oral cancer.

## 5 Conclusion

Multispectral autofluorescence biochemical and metabolic biomarkers of oral cancer were imaged and used as features to develop machine learning models optimized for the discrimination of cancerous and precancerous from benign oral lesions. The outcomes of this study support the potentials of multispectral autofluorescence imaging endoscopy for early detection of oral cancer in a clinical setting. Future research efforts will focus on improving the predictive model development pipeline, ultimately leading to a cost-effective, fast, and reliable clinical diagnostic imaging tool that will facilitate early detection of oral cancer.

## Figures and Tables

**Table 1:** Distribution of the 67 imaged oral lesions based in anatomical location and histopathological diagnosis.

Lesion anatomical location	Histopathology diagnosis				Total number
	Benign	MoD	HiD	SCC	
Buccal mucosa	11	1	1	9	22
Tongue	9	—	—	12	21
Lip	10	—	—	2	12
Gingiva	—	—	2	3	5
Floor of mouth	2	—	—	1	3
Mandible	—	—	—	1	1
Maxilla	—	—	—	1	1
Palate	1	—	—	—	1
Retromolar	1	—	—	—	1
Total number	34	1	3	29	67

MoD: moderate dysplasia; HiD: high-grade dysplasia; SCC: squamous cell carcinoma.

**Table 2:** Summary of autofluorescence spectral intensity features computed per pixel.

	Feature	Total number
Normalized autofluorescence intensity	$I_{390,n}(x,y)$	9
	$I_{452,n}(x,y)$	
	$I_{500,n}(x,y)$	
Autofluorescence intensity ratio	$I_{390}(x,y)∕I_{452}(x,y)$	
	$I_{390}(x,y)∕I_{500}(x,y)$	
	$I_{452}(x,y)∕I_{500}(x,y)$	
	$\left[I_{452}(x,y)+I_{500}(x,y)\right]∕I_{390}(x,y)$	
	$\left[I_{390}(x,y)+I_{500}(x,y)\right]∕I_{452}(x,y)$	
	$\left[I_{390}(x,y)+I_{452}(x,y)\right]∕I_{500}(x,y)$	
Relative features	Δf(x,y)	9
Total number		18

**Table 3:** Classification performance results after leave-one-patient-out-cross-validation for each spectral feature pool and selected feature set size.

	Sensitivity			Specificity		
Spectral feature pool	Top three features	Top four features	Top five features	Top three features	Top four features	Top five features
Absolute (nf = 9)	79%	85%	82%	71%	71%	68%
Relative (nf = 9)	82%	82%	82%	68%	68%	68%
Absolute and relative (nf = 18)	76%	82%	82%	68%	68%	71%

nf: number of features.

**Table 4:** Confusion matrices from the LOPOCV comparing the best performing L1-SVM model against the single-feature L1-SVM model.

		Predicted labels			
		L1-SVM top four features		L1-SVM single feature	
		Benign	Cancer/pre-cancer	Benign	Cancer/pre-cancer
True Labels	Benign (*n* = 34)	24	10	24	10
	MoD (*n* = 1)	0	1	1	0
	HiD (*n* = 3)	0	3	2	1
	SCC (*n* = 29)	5	24	10	19

MoD: moderate dysplasia; HiD: high-grade dysplasia, and SCC: squamous cell carcinoma.

## Statement of Discovery

This study integrated multispectral autofluorescence imaging endoscopy and machine learning to demonstrate automated discrimination of benign from cancerous/pre-cancerous oral lesions. This strategy could potentially lead to a novel clinically compatible computer-assisted detection system for oral cancer screening.
